# Supplementary material for: Empirical methods for controlling false positives and estimating confidence in ChIP-Seq peaks
Source: BMC Bioinformatics. 2008 Dec 5;9:523. doi: 10.1186/1471-2105-9-523 (PMC2628906; doi:10.1186/1471-2105-9-523)
Supplement: Additional File 1 — A variety of html documents from the USeq web site detailing the available applications, their best usage, output file type descriptions, command line menus, etc. [file 1471-2105-9-523-S1.zip › USeqUserGuides/JavaDoc.zip!/JavaDoc/gata/aligner/FileFocusListener.html]

FileFocusListener


|  |  |  |
| --- | --- | --- |
| **Overview** \| **Package** \| **Class** \| **Use** \| **Tree** \| **Deprecated** \| **Index** \| **Help** | |  |
| **PREV CLASS**   **NEXT CLASS** | **FRAMES**    **NO FRAMES**     **All Classes** |  |
| SUMMARY: NESTED \| FIELD \| CONSTR \| METHOD | DETAIL: FIELD \| CONSTR \| METHOD |  |


---


## gata.aligner Class FileFocusListener

```
java.lang.Object
  gata.aligner.FileFocusListener
```

**All Implemented Interfaces:**: java.awt.event.FocusListener, java.util.EventListener

---

``` public class FileFocusListener extends java.lang.Object implements java.awt.event.FocusListener ```

**Author:**
:   nix
    Focus listener firing off upon loss of focus of a text field to check whether the entered file exists

---

**Constructor Summary**

`FileFocusListener()`


| **Method Summary** | |
| --- | --- |
| `void` | `focusGained(java.awt.event.FocusEvent fe)` |
| `void` | `focusLost(java.awt.event.FocusEvent fe)` |
| `void` | `setRefs(javax.swing.JTextField x, javax.swing.JFrame f, javax.swing.JFileChooser c)` |

**Methods inherited from class java.lang.Object**

`equals, getClass, hashCode, notify, notifyAll, toString, wait, wait, wait`

**Constructor Detail**

### FileFocusListener

```
public FileFocusListener()
```


**Method Detail**

### focusGained

```
public void focusGained(java.awt.event.FocusEvent fe)
```

:   **Specified by:**: `focusGained` in interface `java.awt.event.FocusListener`

---


### focusLost

```
public void focusLost(java.awt.event.FocusEvent fe)
```

:   **Specified by:**: `focusLost` in interface `java.awt.event.FocusListener`

---


### setRefs

```
public void setRefs(javax.swing.JTextField x,
                    javax.swing.JFrame f,
                    javax.swing.JFileChooser c)
```


---


|  |  |  |
| --- | --- | --- |
| **Overview** \| **Package** \| **Class** \| **Use** \| **Tree** \| **Deprecated** \| **Index** \| **Help** | |  |
| **PREV CLASS**   **NEXT CLASS** | **FRAMES**    **NO FRAMES**     **All Classes** |  |
| SUMMARY: NESTED \| FIELD \| CONSTR \| METHOD | DETAIL: FIELD \| CONSTR \| METHOD |  |


---
